# The Mitochondrial Genome of *Linichthys laticeps* (Cypriniformes: Cyprinidae): Characterization and Phylogeny

**DOI:** 10.3390/genes14101938

**Published:** 2023-10-14

**Authors:** Renyi Zhang, Tingting Zhu, Hongmei Li, Lei Deng

**Affiliations:** School of Life Sciences, Guizhou Normal University, Guiyang 550025, China

**Keywords:** Labeoninae, *Linichthys laticeps*, mitogenome, phylogenetic relationship

## Abstract

Mitochondrial genomes (mitogenomes) have been widely used in phylogenetic analysis and evolutionary biology. The Labeoninae is the largest subfamily of Cypriniformes and has great economic importance and ecological value. In this study, we sequenced, annotated, and characterized the complete mitogenome of *Linichthys laticeps* and then constructed the phylogenetic tree with previously published Labeoninae mitogenomes. The mitogenome of *L*. *laticeps* was 16,593 bp in length, with an A + T content of 57.1%. The mitogenome contained a standard set of 37 genes and a control region with the same order and orientation of genes as most fish mitogenomes. Each protein-coding gene (PCG) was initiated by an initial ATG codon, excluding *COI*, that began with a GTG codon. Furthermore, most of the PCGs were terminated by a conventional stop codon (TAA/TAG), while an incomplete termination codon (TA/T) was detected in 7 of the 13 PCGs. Most tRNA genes in *L*. *laticeps* were predicted to fold into the typical cloverleaf secondary structures. The *K*_a_/*K*_s_ (ω) values for all PCGs were below one. The phylogenetic relationships of 96 Labeoninae mitogenomes indicated that Labeoninae was not a monophyletic group and *L*. *laticeps* was closely related to the genera *Discogobio* and *Discocheilus*. Overall, our study provided the first complete annotated mitogenome of *L*. *laticeps*, which filled a knowledge gap in Labeoninae and extended the understanding of the taxonomy and mitogenomic phylogeny of the subfamily Labeoninae.

## 1. Introduction

The mitochondrial genome (mitogenome) is one of the most commonly used molecular markers mainly due to its small size, high copy number, matrilineal inheritance, lack of recombination, and high rate of evolution compared to nuclear genome DNA [1,2]. The mitogenome of fish is typically a double-stranded, looped DNA molecule with a size range of 15–18 kb [3,4]. It generally contains 37 genes (22 transfer RNA genes (tRNAs), 13 protein-coding genes (PCGs), and two ribosomal RNA genes (rRNAs)) and one control region (CR) (also known as the D-loop region or the A + T-rich region) [3,4]. Fish mitogenomes have been widely used in fish phylogeny, biogeography, and population genetic structure analyses [5]. During the past 18 years, sequencing and analysis technologies have developed rapidly. A large quantity of fish mitogenomes has been sequenced, annotated, and characterized, covering almost all fish orders [6].

The Labeoninae is the largest and most diverse subfamily in Cyprinidae, including 42 valid genera and over 500 valid species distributed throughout the world [7]. Labeoninae is a small and medium-sized freshwater fish adapted to flowing water, mainly feeding on algae. The species is widely distributed in southern Eurasia and central Africa. Due to special adaptation to the environment, the species of the subfamily have a high diversity in their oral structure. Therefore, taxonomists often use oral structures as key morphological features to identify Labeoninae fishes.

The molecular phylogenetic tree constructed by Yang and Mayden [8] supported that Labeoninae was monophyletic and proposed to subdivide the tribe into two major clades. Then, Zheng et al. [9] divided the Chinese Labeoninae into six clades using the combined molecular data of nuclear genes and mitochondrial genes. The phylogenetic relationship of the subfamily Labeoninae was constructed by Yang et al. [10] using mitochondrial genes and nuclear genes. The results showed that Labeoninae was divided into four clades (Labeoina, Garraina, Osteochilina, and Semilabeoina) with high support based on 142 species from 34 genera of the subfamily. Zhang et al. [11] constructed the phylogenetic trees based on the 12 PCGs of 91 mitogenomes of Labeoninae and divided the subfamily Labeoninae into four major clades. However, the taxonomy and phylogeny of the subfamily Labeoninae have remained a controversial topic for years due to abundant species and morphological diversity.

**Figure 1:**
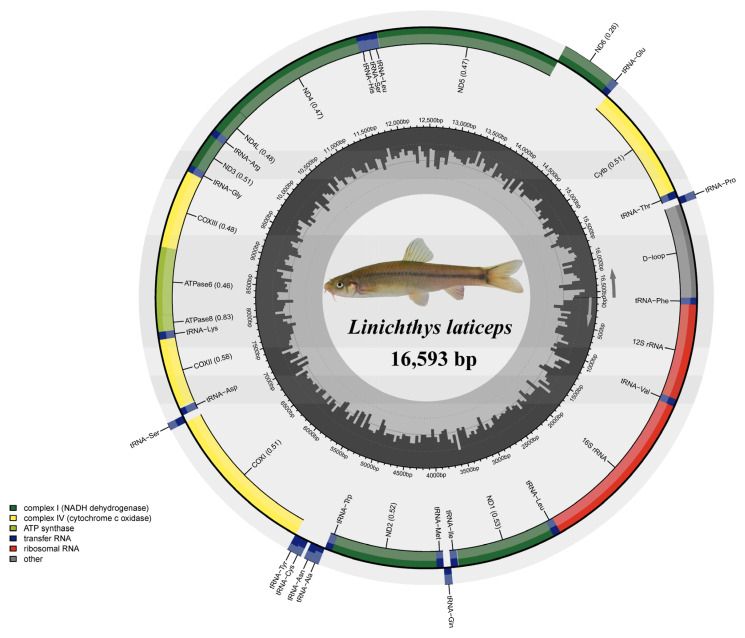
Circular map of the *L*. *laticeps* mitogenome.

*L*. *laticeps* (Lin & Zhang, 1986), originally named *Barbodes laticeps* by Lin and Zhang, is mainly distributed in the Nanming River and the Maling River in Guiyang, China [12,13]. They live mainly in mountain streams and the outlet of the subterranean river. In 2005, *B*. *laticeps* was renamed *L*. *laticeps* based on morphological data, and a new genus, *Linichthys,* was established based on it [12]. At present, there is only one species in the genus. The main characteristic of *L*. *laticeps* is a shallow depression in the upper lip, the lower lip being horseshoe-shaped without a sharp cuticle, and a black vertical line above the lateral line of the body (Figure 1). To date, there is no record of the complete sequence of the mitogenome of the *Linichthys* genus in the National Center for Biotechnology Information (NCBI).

In this study, we newly sequenced, assembled, and characterized the complete mitogenome of *L*. *laticeps*. Specifically, we analyzed the characteristics of mitogenome size, mitogenome structure, organization, nucleotide composition, codon usage, secondary structures of tRNAs, and evolutionary rates. Finally, the phylogenetic position of *L*. *laticeps* within Labeoninae, as well as the relationship of the subfamily Labeoninae, was defined based on 13 PCGs. The new mitogenome data will lay the foundation for the phylogenetic analysis and taxonomy of Labeoninae.

## 2. Materials and Methods

### 2.1. Sampling, DNA Extraction, and High-Throughput Sequencing

Specimens of *L*. *laticeps* were collected from the Maling River, Huaxi District, Guiyang City, Guizhou Province, China, in August 2022. Samples were conserved in absolute ethanol and then stored in a −20 °C freezer. Total genomic DNA was isolated from the muscle of a specimen with the DNeasy Blood & Tissue Kit (Qiagen Inc., Hilden, Germany). The genomic DNA was fragmented by a Covaris Ultrasonic Process. Then, the DNA library was completed by terminal repair, A-tail addition, sequencing adaptor addition, purification, and PCR amplification. The concentration of the library was checked with Qubit 2.0. The inserted fragments of the library were detected by Agilent 2100. Sequencing was performed using an Illumina sequencing platform by the DNA Stories Bioinformatics Center (Chengdu, Sichuan, China).

### 2.2. Sequence Assembly, Annotation, and Analysis

The mitogenome was assembled using GetOrganelle v. 1.7.7.0 [14]. After assembly, the complete mitogenome was annotated using MitoAnnotator 3.94 [6]. The annotated tRNA genes were reconfirmed with the tRNAscan-SE server v. 1.21 [15]. The online tool MITOS Web Server was used to predict tRNA second structures [16]. The formulae A + T skew = (A − T)/(A + T) and G + C skew = (G − C)/(G + C) were used to calculate the strand asymmetry of the mitogenome sequence [17]. The nucleotide composition and relative synonymous codon usage (RSCU) of the mitogenome sequence were determined by MEGA 6 [18]. 

The substitution rates of the different PCGs were obtained among closely related species. The software DnaSP 6.0 [19] was used to calculate the values of *K*_a_ (the nonsynonymous substitution rate), *K*_s_ (the synonymous substitution rate), and ω (the *K*_a_/*K*_s_ ratio).

### 2.3. Phylogenetic Analysis

The phylogenetic relationships of 96 species representing 29 genera of Labeoninae were reconstructed (Table S1). *Cyprinus carpio*, *Danio rerio*, and *Squalius lepidus* were chosen as the outgroups for the construction of the phylogenetic tree (Table S1). PhyloSuite v1.2.3 [20] was used to extract the complete mitogenome genes. A batch alignment of 13 PCG sequences from 99 species was performed using MAFFT v7.0 [21] integrated into PhyloSuite. Phylogenetic analyses were performed using both the maximum likelihood (ML) and Bayesian inference (BI) methods. PartitionFinder2 [22], with the greedy algorithm and the modified Akaike information criterion (AICc), was used to select the best-fit partitioning scheme and evolutionary model for 39 predefined partitions. The ML phylogenetic tree was constructed with IQ-TREE v1.6.12 [23] under an edge-linked partition model for 5000 ultrafast bootstraps [24], as well as the Shimodaira–Hasegawa-like approximate likelihood-ratio test [25]. MrBayes 3.2.6 [26] was used to perform the BI analysis under two independent Markov chain Monte Carlo (MCMC) runs with four chains each that were simultaneously conducted for five million generations. The initial 25% of trees from each MCMC chain run were discarded as burn-in. The online tool Interactive Tree Of Life (iTOL) (https://itol.embl.de/) (accessed on 25 August 2023) was used to visualize, annotate, and manage the phylogenetic trees.

## 3. Results and Discussion

### 3.1. Genome Organization and Base Composition

In this study, the complete mitogenome of *L*. *laticeps* was sequenced, assembled, and annotated (GenBank accession number: OR343919). The complete mitogenome sequence was 16,593 bp in length. It consisted of 37 genes (2 rRNAs, 22 tRNAs, and 13 PCGs) and a control region (Figure 1; Table 1). Among these genes, 28 genes were encoded on the heavy strand, and the other nine genes were encoded on the light strand (Figure 1; Table 1). Our analysis comparing the mitogenome of the species showed that the mitogenome size, gene numbers, and gene arrangement were highly conserved, which was consistent with other published mitogenomes of Labeoninae [11,27].

The composition of the A, T, G, and C nucleotides of *L*. *laticeps* was 31.4%, 25.7%, 16.0%, and 26.9%, respectively. The A + T content was 57.1%, showing a relatively slight A + T bias (Table 2). The control region had the highest A + T content, reaching 66.7%. In contrast, the first codon position of PCGs was the region with the lowest A + T content, which was 49.0%. The nucleotide composition was consistent with that of the Labeoninae genomes (Table S1). The values of the A + T and G + C skews were a measure of compositional asymmetry [28]. The A + T skew and the G + C skew were 0.100 and −0.254, respectively, in the mitogenome of *L*. *laticeps*. Fish mitogenomes usually tend to have the characteristics of A + T bias [4,5].

In the *L*. *laticeps* mitogenome, there were 14 intergenic spacers ranging from 1 bp to 33 bp in length. The two long intergenic spacers were located between *tRNA^Asn^* and *tRNA^Cys^* (33 bp) and *tRNA^Asp^* and *COII* (13 bp). Gene overlaps in the mitogenome were found at five locations, and their total size was 21 bp. The minimum overlapped region was 1 bp, and the maximum overlapped region was 7 bp. The mitochondrial gene overlap and the gene spacer have long been known throughout teleost species [29,30].

### 3.2. Protein-Coding Genes and Codon Usage

The total length of the 13 PCGs in *L*. *laticeps* was 11,400 bp (Table 2). Among these PCGs, only *NAD6* was located on the light strand, while the remaining 12 PCGs were encoded on the heavy strand. The A + T content of the PCGs was 57.0%. The start codon of most PCGs was ATG, but *COI* started with GTG. The GTG start codon for *COI* was presented in the mitogenomes of many fish species [4,5,29]. The standard stop codon (TAA) was used by six genes (*ND1*, *COI*, *ATPase8*, *ND4L*, *ND5*, and *ND6*), and two incomplete stop codons (T and TA) were used by seven genes (*ND2*, *COII*, *ATPase6*, *COIII*, *ND3*, *ND4*, and *Cyt b*). The incomplete stop codon was commonly found in vertebrate mitogenomes, which was presumed to be completed by post-transcriptional modification, such as polyadenylation [31].

**Figure 2:**
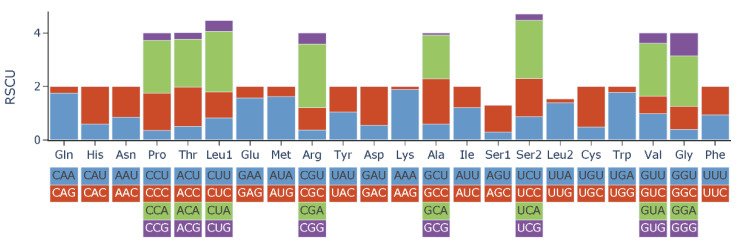
Relative synonymous codon usage (RSCU) of 13 PCGs in the mitogenome of *L*. *laticeps*.

The RSCU values of *L*. *laticeps* are shown in Table 3 and Figure 2. The 13 PCGs expressed a total of 3794 amino acid triplets, excluding the stop codon. The highest number of amino acids was Leu, followed by Ala, Thr, Ile, and Phe. Cys was the lowest at 25. The codon usage of PCGs was estimated based on RSCU values. The results showed that the most frequent codons among the 13 PCGs were CUA of Leu, AUU of Ile, and UUA of Leu.

### 3.3. Evolutionary Rates and Patterns

**Figure 3:**
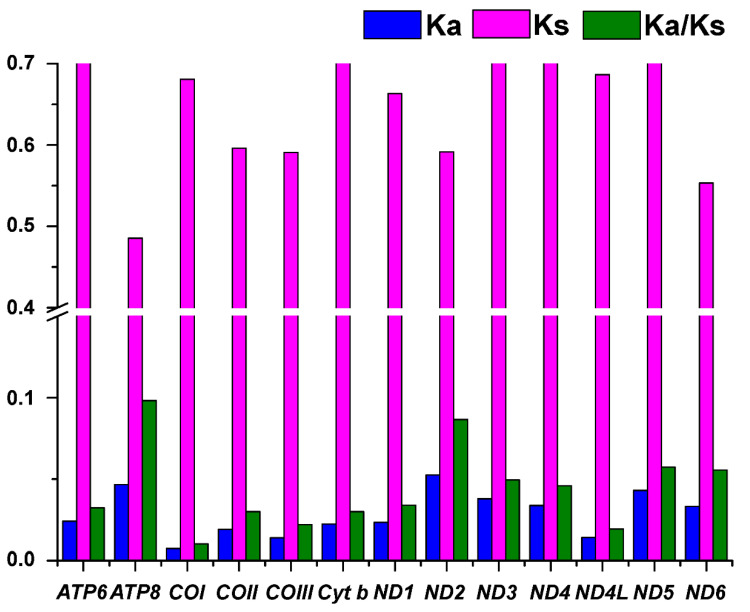
*K*_a_, *K*_s_, and ω values of 13 PCGs in *L*. *laticeps*.

To better understand the evolutionary patterns of the 13 PCGs and the role of selection, we calculated the values of *K*_a_, *K*_s_, and ω for each protein-coding gene (Figure 3). The results showed that ND4L has the lowest *K*_a_ value (0.486), and ND3 has the highest *K*_a_ value (0.78). COI has the lowest *K*_s_ value (0.007), and ND2 has the highest *K*_s_ value (0.052). The average ω value was 0.043, ranging from 0.01 (COI) to 0.098 (AT8). The ω values for all PCGs were well below one, suggesting that these functional genes evolved under purifying selection [32].

### 3.4. Ribosomal and Transfer RNA Genes

The ribosomal RNA gene encoded ribosomal RNA, which was the essential component of the ribosome and was involved in the protein synthesis processes. In the *L*. *laticeps* mitogenome, the sequence length of the small (12S) rRNA and large (16S) rRNA genes was 954 bp and 1683 bp, respectively. The two RNA genes were located close together, between the *tRNA^Phe^* and *tRNA^Leu(UUR)^* genes, and split by the *tRNA^Val^* gene. The A+T content of the two RNA genes was 55.0%. Furthermore, the concatenated nucleotide sequence of two rRNA genes exhibited a positive A + T skew (0.276) and a negative G + C skew (−0.089) in *L*. *laticeps*.

**Figure 4:**
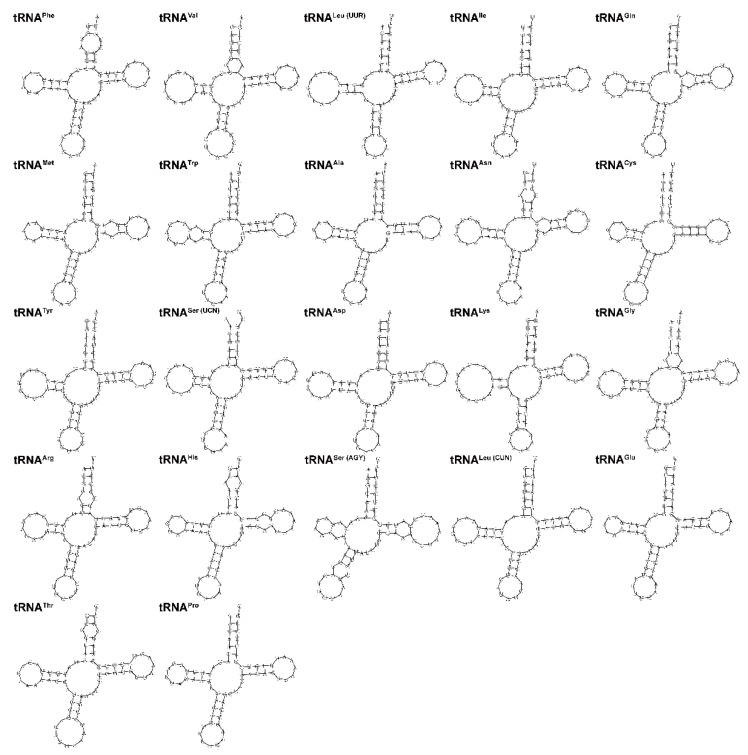
The secondary structures of 22 tRNA genes in the *L*. *laticeps* mitogenome were predicted by MITOS Web Server.

Transfer RNA was one of the classical non-coding RNAs and was often referred to as tRNA. The secondary structures of 22 tRNA genes are shown in Figure 4. We found that only *tRNA^Ser(AGY)^* lacked the dihydrouridine (DHU) arm, and the remaining tRNA genes all formed a typical cloverleaf secondary structure. This loss was reported in many fishes [30]. The tRNA genes were distributed throughout the mitogenome and ranged in length from 67 bp (*tRNA^Cys^*) to 76 bp (*tRNA^Leu(UUR)^* and *tRNA^Lys^*). The concatenated nucleotide sequence of the 22 tRNAs showed a high A+T bias, accounting for 55.5%, and exhibited a positive A + T skew (0.045) and a positive G + C skew (0.043) (Table 2).

### 3.5. Control Region

The non-coding region in the mitogenome was usually called the control region. Because this region contains promoters, it was critical for the initiation of replication and transcription in vertebrates [33,34]. The control region of *L*. *laticeps* was placed after *tRNA^Pro^*, with a sequence length of 934 bp. The A + T skew was positive (0.001), and the G + C skew was negative (−0.189), suggesting a preference for using A and C bases in the control region.

### 3.6. Phylogenetic Analysis

**Figure 5:**
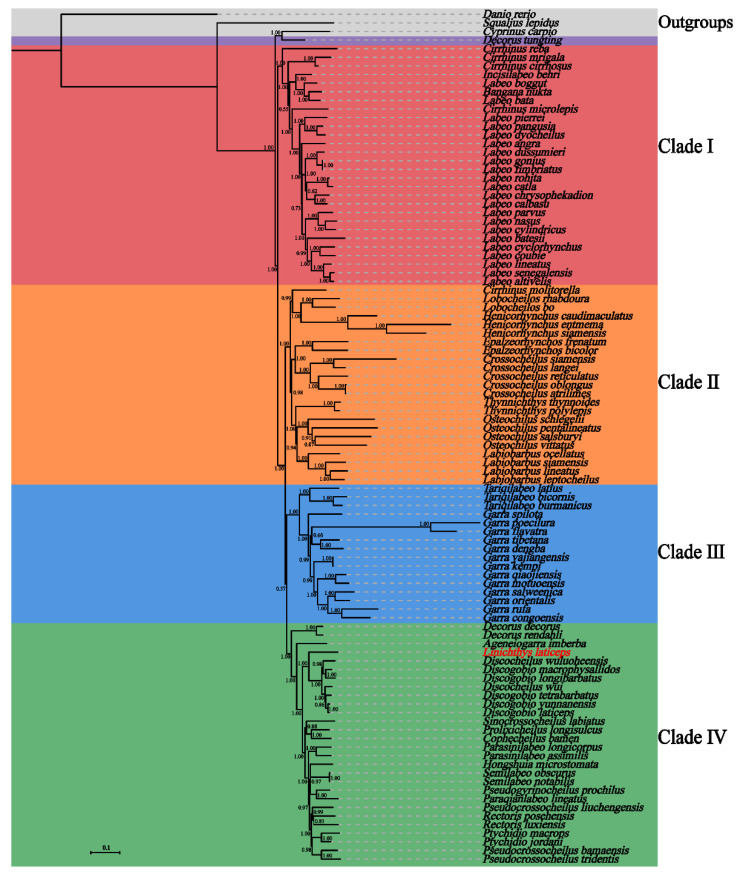
Bayesian inference phylogenetic tree inferred based on the 13 PCGs of *L*. *laticeps* and other species within the subfamily Labeoninae. The number at each node indicates the posterior probabilities. The detailed species information used for the phylogenetic analyses is provided in Table S1.

The phylogenetic analysis based on ML and BI was performed using the nucleotide sequences of 13 PCGs of *L*. *laticeps* and the other 96 species of the Labeoninae subfamily (Figure 5 and Figure S1). The results showed that the topological structures of the ML tree and the BI tree were similar (Figure 5 and Figure S1), and the 96 species were grouped into four clades except for *Decorus tungting*. The phylogenetic positions of clade II and clade III were reversed in the BI and ML trees, indicating that the evolutionary relationship of these two clades in the Labeoninae was unclear. Overall, the phylogeny of Labeoninae reconstructed in our study was very similar to previous studies [11,35]. However, the monophyly of Labeoninae has not been confirmed, and the intergeneric relationship of the labeoninae was controversial. *D*. *tungting* was previously known as *Bangana tungting*, and *Bangana* was composed of two clades in Labeoninae in previous molecular phylogenetic studies [9]. In recent years, the genus *Bangana* has been revised and renamed into a new genus, *Decorus* [36]. However, the phylogenetic position of *D*. *tungting* in this study was different from that in the previous study [36]. Therefore, we suggest that the taxonomy of *D*. *tungting* requires further revision.

Clade I includes *Cirrhinus*, *Incisilabeo*, *Labeo*, and *Bangana*. The group was located at the basic position of Labeoninae in the phylogenetic tree. All *Labeo* species were clustered in Clade I, but they did not form a monophyletic group (Figure 5 and Figure S1). Previous studies have also confirmed that *Labeo* was not a monophyletic group [10]. Clade II included *Cirrhinus*, *Crossocheilus*, *Epalzeorhynchos*, *Henicorhynchus*, *Labiobarbus*, *Lobocheilos*, *Osteochilus*, and *Thynnichthys*. Except for *Cirrhinus molitorella*, the other genera in Clade II formed their monophyletic groups. All species of the genus *Cirrhinus,* except *C*. *molitorella,* were located in Clade I. Clade III included *Garra* and *Tariqilabeo*. Within this clade, *Tariqilabeo* and *Garra* both formed their clades with high bootstrap support. Clade IV included *Ageneiogarra*, *Cophecheilus*, *Decorus*, *Discogobio*, *Discocheilus*, *Hongshuia*, *Linichthys*, *Paraqianlabeo*, *Parasinilabeo*, *Pseudogyrinocheilus*, *Pseudocrossocheilus*, *Prolixicheilus*, *Ptychidio*, *Rectoris*, *Semilabeo*, and *Sinocrossocheilus*. There were 16 genera in Clade IV, and the newly sequenced *L*. *laticeps* was confirmed as a member of the subfamily Labeoninae in this clade. The mitogenome of *L*. *laticeps* was most closely related to the genera *Discogobio* and *Discocheilus*. This was consistent with the phylogenetic relationships based on the combined mitochondrial and nuclear gene datasets [10]. 

In recent years, many genera of Labeoninae have been taxonomically revised, while new genera have been constantly added. However, due to the diversity and complexity of the morphology, there were many practical classification problems for some groups. The phylogenetic tree revealed that many genera were non-monophyletic, such as *Cirrhinus*, *Labeo*, and *Pseudocrossocheilus*, which conflicted with the past taxonomy based on morphology. The results indicated that the validities of some traditional genera required further checks.

## 4. Conclusions

Herein, we first described the complete mitogenome of *L*. *laticeps* from the subfamily Labeoninae. The complete mitogenome length was 16,593 bp, including 13 PCGs, two rRNAs, 22 tRNAs, and one control region, with the same mitogenome structure as other teleosts. The phylogenetic analysis of Labeoninae revealed that Labeoninae was not a monophyletic group, and *L*. *laticeps* was closely related to the genera *Discogobio* and *Discocheilus*. The results advanced our understanding of the phylogenetic relationship of Labeoninae. In addition, the mitogenomic sequence data presented here will contribute to further taxonomical study, population genetics, and the phylogeography of *L*. *laticeps*.

## Figures and Tables

**Table 1:** Organization of the mitogenome of *L*. *laticeps*.

Gene	Location		Size (bp)	Codon		Anticodon	Strand *	Intergenetic Nucleotides
	From	To		Start	Stop			
*tRNA^Phe^*	1	69	69			GAA	H	0
*12S rRNA*	70	1023	954				H	0
*tRNA^Val^*	1024	1095	72			TAC	H	0
*16S rRNA*	1096	2778	1683				H	0
*tRNA^Leu(UUR)^*	2779	2854	76			TAA	H	0
*ND1*	2856	3830	975	ATG	TAA		H	1
*tRNA^Ile^*	3836	3907	72				H	5
*tRNA^Gln^*	3906	3976	71			GAT	L	−2
*tRNA^Met^*	3978	4046	69			TTG	H	1
*ND2*	4047	5091	1045	ATG	T--	CAT	H	0
*tRNA^Trp^*	5092	5162	71				H	0
*tRNA^Ala^*	5165	5233	69			TCA	L	2
*tRNA^Asn^*	5235	5307	73			TGC	L	1
*tRNA^Cys^*	5341	5407	67			GTT	L	33
*tRNA^Tyr^*	5409	5479	71			GCA	L	1
*COI*	5481	7031	1551	GTG	TAA	GTA	H	1
*tRNA^Ser(UCN)^*	7032	7102	71				L	0
*tRNA^Asp^*	7106	7177	72			TGA	H	3
*COII*	7191	7881	691	ATG	T--	GTC	H	13
*tRNA^Lys^*	7882	7957	76				H	0
*ATPase8*	7959	8123	165	ATG	TAA	TTT	H	1
*ATPase6*	8117	8799	683	ATG	TA-		H	−7
*COIII*	8800	9584	785	ATG	TA-		H	0
*tRNA^Gly^*	9585	9656	72				H	0
*ND3*	9657	10,005	349	ATG	T--	TCC	H	0
*tRNA^Arg^*	10,006	10,075	70				H	0
*ND4L*	10,076	10,372	297	ATG	TAA	TCG	H	0
*ND4*	10,366	11,746	1381	ATG	T--		H	−7
*tRNA^His^*	11,747	11,815	69				H	0
*tRNA^Ser(AGY)^*	11,816	11,884	69			GTG	H	0
*tRNA^Leu(CUN)^*	11,886	11,958	73			GCT	H	1
*ND5*	11,962	13,785	1824	ATG	TAA	TAG	H	3
*ND6*	13,782	14,303	522	ATG	TAA		L	−4
*tRNA^Glu^*	14,304	14,372	69				L	0
*Cyt b*	14,378	15,518	1141	ATG	T--		H	5
*tRNA^Thr^*	15,519	15,590	72				H	0
*tRNA^Pro^*	15,590	15,659	70				L	−1
control region	15,660	16,593	934				H	0

* H—heavy strand; L—light strand.

**Table 2:** Nucleotide composition (%) and skewness of the *L*. *laticeps* mitogenome.

Location	Size (bp)	A	T	G	C	A + T	G + C	A + T Skew	G + C Skew
Genome	16,593	31.4	25.7	16.0	26.9	57.1	42.9	0.100	−0.254
PCGs	11,400	29.3	27.7	15.5	27.5	57.0	43.0	0.028	−0.279
1st codon position	3800	27.0	22.0	25.7	25.3	49.0	51.0	0.102	0.008
2nd codon position	3800	18.5	40.7	13.6	27.2	59.2	40.8	−0.375	−0.333
3rd codon position	3800	42.3	20.4	7.3	30.0	62.7	37.3	0.349	−0.609
rRNA	2637	35.1	19.9	20.5	24.5	55.0	45.0	0.276	−0.089
tRNA	1563	29.0	26.5	23.2	21.3	55.5	44.5	0.045	0.043
control region	934	33.4	33.3	13.5	19.8	66.7	33.3	0.001	−0.189

**Table 3:** Codon number and RSCU of 13 PCGs in the mitogenome of *L*. *laticeps*.

Amino Acid	Codon	Count	RSCU	Amino Acid	Codon	Count	RSCU
Phe	UUU	108	0.94	Tyr	UAU	59	1.04
Phe	UUC	123	1.06	Tyr	UAC	54	0.96
Leu	UUA	145	1.39	stop codon	UAA	6	4
Leu	UUG	16	0.15	stop codon	UAG	0	0
Leu	CUU	85	0.82	His	CAU	32	0.59
Leu	CUC	102	0.98	His	CAC	76	1.41
Leu	CUA	235	2.26	Gln	CAA	88	1.74
Leu	CUG	42	0.4	Gln	CAG	13	0.26
Ile	AUU	178	1.21	Asn	AAU	52	0.85
Ile	AUC	116	0.79	Asn	AAC	70	1.15
Met	AUA	144	1.62	Lys	AAA	72	1.89
Met	AUG	34	0.38	Lys	AAG	4	0.11
Val	GUU	54	0.99	Asp	GAU	21	0.55
Val	GUC	35	0.64	Asp	GAC	55	1.45
Val	GUA	109	1.99	Glu	GAA	80	1.57
Val	GUG	21	0.38	Glu	GAG	22	0.43
Ser	UCU	33	0.86	Cys	UGU	6	0.48
Ser	UCC	55	1.44	Cys	UGC	19	1.52
Ser	UCA	83	2.17	Trp	UGA	106	1.78
Ser	UCG	9	0.24	Trp	UGG	13	0.22
Pro	CCU	19	0.36	Arg	CGU	7	0.37
Pro	CCC	74	1.38	Arg	CGC	16	0.84
Pro	CCA	106	1.98	Arg	CGA	45	2.37
Pro	CCG	15	0.28	Arg	CGG	8	0.42
Thr	ACU	39	0.51	Ser	AGU	11	0.29
Thr	ACC	112	1.46	Ser	AGC	38	1
Thr	ACA	137	1.79	stop codon	AGA	0	0
Thr	ACG	19	0.25	stop codon	AGG	0	0
Ala	GCU	49	0.59	Gly	GGU	24	0.39
Ala	GCC	140	1.69	Gly	GGC	54	0.87
Ala	GCA	136	1.64	Gly	GGA	116	1.88
Ala	GCG	7	0.08	Gly	GGG	53	0.86

## Data Availability

The mitogenome sequence data are openly available in GenBank of NCBI under accession no. OR343919.

## Supplementary Materials

The following supporting information can be downloaded at https://www.mdpi.com/article/10.3390/genes14101938/s1. Table S1: Taxon information of the subfamily Labeoninae and three outgroup species analyzed in this study. Figure S1: Maximum likelihood phylogeny based on the concatenated nucleotide sequences of 13 PCGs showed the phylogenetic relationship of *L*. *laticeps* with other Labeoninae species. The number on each node indicates the ML bootstrap values.
