# Supplementary figures and images for: The Mitochondrial Genome of Linichthys laticeps (Cypriniformes: Cyprinidae): Characterization and Phylogeny
Source: Genes (Basel). 2023 Oct 14;14(10):1938. doi: 10.3390/genes14101938 (PMC10606506; doi:10.3390/genes14101938)

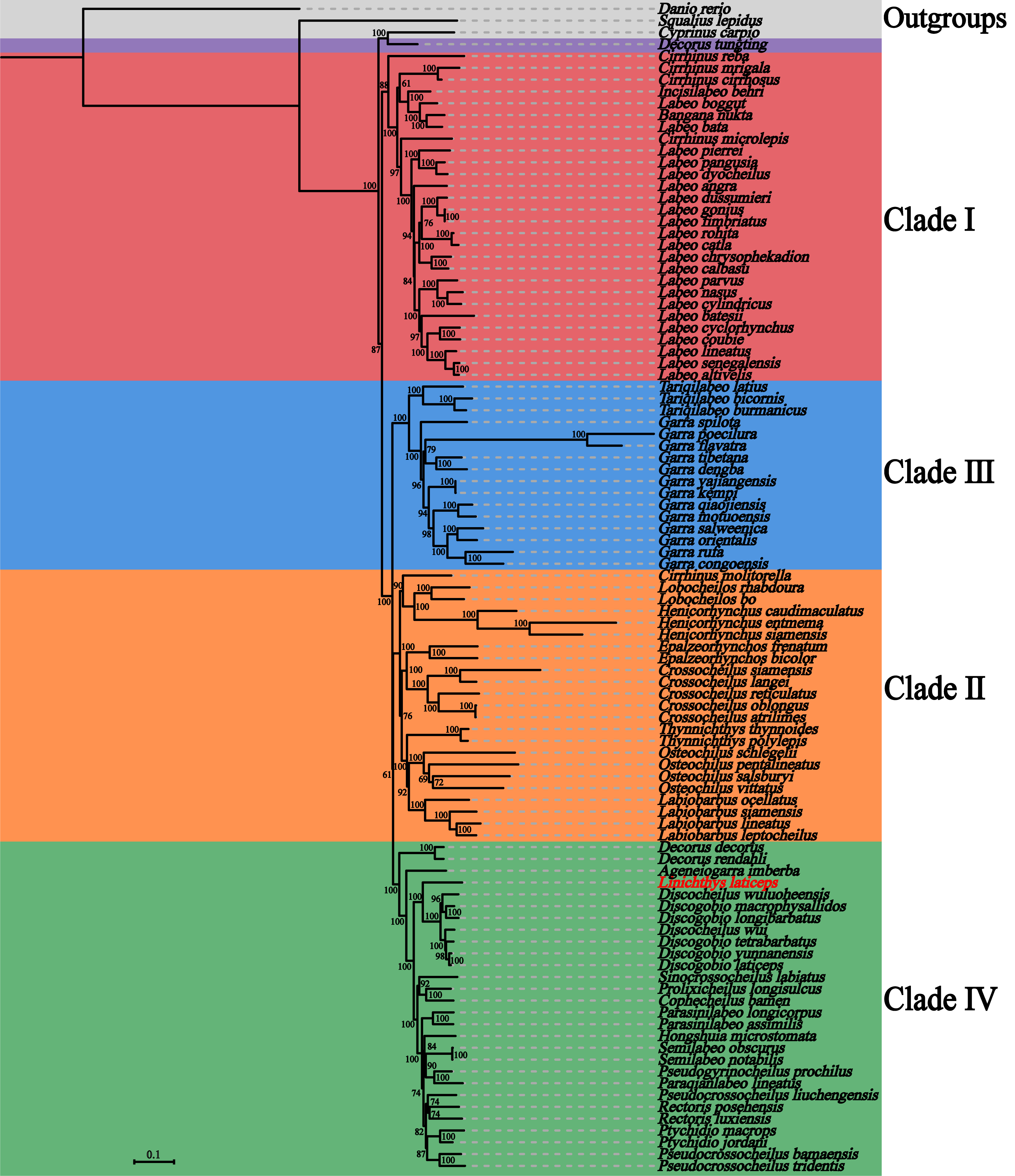

Supplement: Supplementary file 1 [file genes-14-01938-s001.zip › Figure S1.tif]
